# Does the Phase-One Functional Therapy Increase the Risk of an External Apical Root Resorption Following the Phase-Two Fixed Orthodontic Treatment? A Pilot Study

**DOI:** 10.3390/dj13030095

**Published:** 2025-02-24

**Authors:** Sara Eslami, Jakob Stuhlfelder, Suh-In Rhie, Sarah Bühling, Mauricio Gonzalez Balut, Ludovica Nucci, Abdolreza Jamilian, Babak Sayahpour

**Affiliations:** 1 Department of Orthodontics, Johann-Wolfgang Goethe University, 60596 Frankfurt am Main, Germany; stuhlfelder@med.uni-frankfurt.de (J.S.); buehling@med.uni-frankfurt.de (S.B.); sayahpour@med.uni-frankfurt.de (B.S.); 2 MIRKHYL DENTALWELT, Bad Vilbel Frankfurter Straße 47, Bad Vilbel, 61118 Frankfurt am Main, Germany; 3 Department of Orthodontics and Dentofacial Orthopedics, Loma Linda University, San Bernadino, CA 92408, USA; mgbalut@hotmail.com; 4 Multidisciplinary Department of Medical-Surgical and Dental Specialties, University of Campania Luigi Vanvitelli, 80131 Naples, Italy; ludortho@gmail.com; 5 Department of Orthodontics, Dental School, Tehran Islamic Azad University of Medical Sciences, Tehran 19395-1495, Iran; info@jamilian.net; 6 City of London Dental School, University of Greater Manchester, Bolton BL3 5AB, UK

**Keywords:** root resorption, orthodontic appliances, fixed, functional

## Abstract

**Background**: This retrospective study aimed to analyze the frequency and extent of apical root resorptions (EARR) during orthodontic treatment in the upper and lower incisors, as well as lower molars, using orthopantomograms (OPG). Potential influencing factors such as age, gender, root shape, type of orthodontic appliance, and treatment duration were examined as well. **Methods**: A total of 57 patients who completed their treatment at the orthodontic department of the Goethe University of Frankfurt between 2011 and 2018 were included in the study. These patients had a combined total of 570 teeth, which were divided into two groups. Group 1 consisted of 20 patients (average age at T0: 10.1 ± 1.2 years old) received a one-phase fixed orthodontic treatment using passive self-ligating Damon bracket system (average duration of 2.1 years ± 6 months), while group 2 consisted of 37 patients (average age at T0: 12.4 ± 2.8 years old) underwent a two-phase therapy, which involved a phase-one functional therapy (average duration of 1.7 years ± 6 months) prior to the phase-two fixed orthodontic treatment with the Damon system (average duration of 1.5 ± 4 months) with a total treatment time of 3.2 years ± 7 months. To determine the extent of post-treatment root resorption of the upper and lower incisors, as well as the first lower molars, crown–root ratio was calculated for each tooth using the pre- and post-treatment OPGs. Additionally, each tooth was assigned a degree of resorption according to the Levander and Malmgren classification. The inter-group comparisons were conducted using the Wilcoxon Mann–Whitney U test. Spearman’s correlation analysis was used to assess the relationship between age, treatment duration, and EARR. The association between gender, root morphology, and EARR was evaluated using the Wilcoxon Mann–Whitney U test. For nominally scaled variables, the Chi-square test was used. The statistical significance was set at *p* < 0.05. **Results**: No statistically significant differences were seen between groups 1 and 2 regarding the degree of root resorption (*p* = 0.89). The study found that the average root resorption for all examined teeth was −5.14%, indicating a slight reduction in the length of the tooth roots after orthodontic treatment. However, no significant differences were observed concerning gender, age, type of orthodontic appliance or treatment duration. Comparisons between upper and lower jaws also did not yield statistically significant differences. The majority of teeth in the study exhibited a normal root shape. The short root length and a pipette formed roots were significantly associated with a higher risk of root resorption (*p* = 0.001). **Conclusions**: The study’s findings suggest that the two-phase orthodontic treatment does not increase the risk of EARR compared to one-phase therapy significantly. Some degree of root resorption occurred as a result of orthodontic treatment in both groups. Notably, abnormal root forms were identified as influential factors that could help predict the likelihood of root resorption following orthodontic treatment.

## 1. Introduction

External apical root resorption (EARR) is commonly discussed as a consequence of orthodontic treatment. Throughout life, localized root resorption is a natural and constant remodeling process, which is a response to oral microtraumas. Typically, roots do not naturally shorten with age unless they are subjected to excessive forces like bruxism or tongue thrusting, which can compress the periodontal ligament. Under normal circumstances, the body can undergo appositional repair to correct resorptive defects. However, irreversible root shortening occurs when there are excessive forces applied or decreased resistance to normal forces [1].

During orthodontic treatment, the periodontium experiences different forces that facilitate tooth movement. Specific remodeling mechanisms must occur in order to enable the orthodontic tooth movement. Tension and compression forces play a key role in enabling this movement [2,3]. Bone formation occurs on the tension side, while bone resorption takes place on the compression side. This resorption is mediated by osteoclast activation, which is triggered by proinflammatory cytokines that upregulate the receptor activator of nuclear factor-κB ligand (RANKL). RANKL binds to its receptor, RANK, to promote osteoclastogenesis. Excessive compressive forces generated during orthodontic treatment can markedly increase RANKL expression in periodontal tissues, potentially leading to orthodontically induced external root resorption (ERR). This may result from undermining resorption, a process in which excessive forces disrupt blood flow, causing localized hyalinization of the periodontal ligament, thereby delaying direct alveolar-bone resorption and slowing tooth movement. This ischemia-induced process leads to the temporary disappearance of osteocytes and necrosis of the affected tissue, which must first be removed before resorption and remodeling can resume. Direct resorption is associated with lower forces, whereas higher forces increase the likelihood of undermining resorption, making precise force control critical in preventing ERR [4]. If this resorption extends to the dentin, it becomes irreversible [5,6,7]. Therefore, it is essential to avoid excessive force application, as it may disrupt the balance of alveolar-bone conversion processes, leading to tilting towards the resorptive side and causing external apical root resorption. Root resorption of permanent teeth is a pathological process that can lead to serious consequences, depending on its severity [8,9].

The aim of this study was to examine potential factors contributing to EARR. The study centered on evaluating the influence of distinct orthodontic treatment approaches. This encompassed comparing a phase-one treatment involving only fixed orthodontic appliances with a phase-two approach, which incorporated functional orthodontic appliances prior to the application of fixed appliances. Additionally, the study analyzed variables such as treatment duration, gender, age and root shape to determine their effects.

## 2. Materials and Methods

This study received approval from the Ethics Committee at Goethe University in Frankfurt, Germany (identifier 20-823, 29 July 2020). The sample-selection process was retrospective, using records from the orthodontics department. The selection was solely based on the assessment of initial and final root resorption in the upper and lower incisors, as well as the first lower molars, using orthopantomograms (OPG) taken before and after orthodontic treatment. The study encompassed a total of 57 patients, whose treatments began between 1 January 2011 and 31 December 2018. Only patients, who underwent orthodontic treatment at the Orthodontic Department of the Goethe University of Frankfurt with high-quality, easily assessable before-and-after OPGs were included in the present study. Patients receiving orthodontic therapy other than fixed orthodontic appliances or removable functional appliances were excluded from the sample. The presence of radiological indications of pre-existing root resorption or pathological/inflammatory reactions on OPGs, as well as previous endodontical treatments of incisors and lower molars, led to exclusion of the samples. Furthermore, patients with a history of oral, maxillofacial surgery, trauma, systemic diseases affecting bone metabolism (e.g., osteoporosis), enamel formation disorders, periodontal diseases, and signs and symptoms of craniomandibular disorders were not considered for this study. Furthermore, dental crowns, extensive dental restorations, or the recontouring and malformations of incisors and lower molars led to exclusion of the samples.

The sample was divided into two groups: Group 1 comprised 20 patients (11 female, 9 male) with an initial age of 10.1 years, who underwent exclusively fixed orthodontic treatment. Group 2 consisted of 37 patients (17 female, 20 male) with an initial age of 12.4 years, and they underwent a phase-two therapy involving removable functional orthodontic appliances before proceeding to fixed orthodontic treatment. The fixed orthodontic treatment of both groups was carried out using Damon-Q self-ligating brackets (Ormco, Orange, CA, USA) with standard values, 0.022-in slots, and the following archwire sequence: 0.014 CuNiTi Damon; 0.016 CuNiTi Damon; 0.016 × 0.025 CuNiTi Damon; 0.018 × 0.025 CuNiTi Damon; 0.019 × 0.025 SS. In total, 570 teeth were assessed, with 200 teeth belonging to group 1 and 370 teeth belonging to group 2.

**Figure 1:**
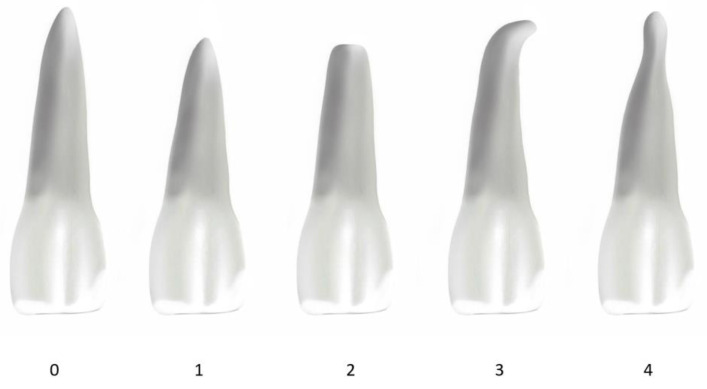
Root-shape scoring system.

**Figure 2:**
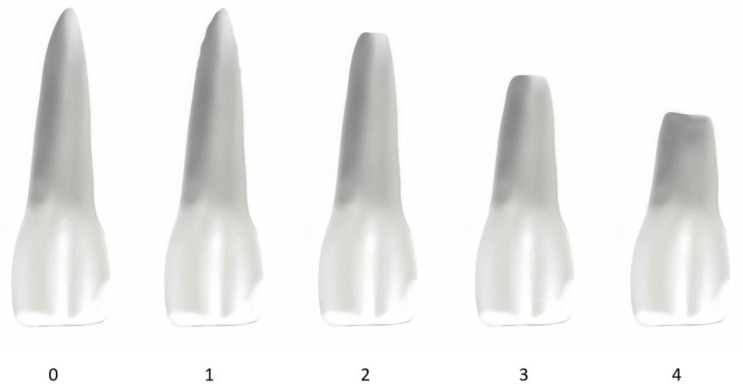
Root-resorption grading system.

The patient records were used to gather relevant information for this study, such as the patient’s gender, date of birth, and the date of the beginning and end of their treatment. Their medical history was referenced to confirm any potential exclusion criteria. The treatment process was examined based on when it started and ended. By merging these specifics with the patient’s birthdate, accurate calculations were performed to figure out the complete treatment duration and the patient’s age at the beginning of the treatment. The assessment of root shape and apical root resorption was executed using Levander and Malmgren’s classifications [10]. The configuration of the incisors and lower first molars’ roots was observed through the analysis of pre- (T1) and post- (T2) treatment OPGs, and subsequently assigned scores ranging from 0 to 4 (Figure 1). A similar procedure was followed for the evaluation of root resorption, wherein the extent of resorption in the teeth was appraised and assigned grades between 0 and 4 (Figure 2).

The categorization of root shapes is as follows:

Score 0: normal root;

Score 1: short root;

Score 2: blunt root;

Score 3: root with apical bend;

Score 4: pipette-shaped root.

The grading system for evaluating root resorption includes the following categories:

Grade 0: absence of root resorption;

Grade 1: mild resorption, root of normal length but irregular contour;

Grade 2: moderate resorption, apex with almost straight contour;

Grade 3: accentuated resorption, loss of up to 1/3 of the root length;

Grade 4: extreme resorption, loss of more than 1/3 of the root length.

Additionally, a calculation of the crown–root ratio (CRR) was conducted. A digital analysis of OPGs was performed using OnyxCeph3TM (Image Instruments, Chemnitz, Germany) and repeated by the same observer after a 2-week interval to ensure intra-rater reliability, which yielded a good intraclass correlation coefficient (ICC > 0.90). To ensure the high inter-rater reliability, a separate evaluator participated in the assessment of the CRR (ICC > 0.75). Upon detection of samples with ICC < 0.90, the measurements were reviewed and resolved until a score of above 0.90 was reached. The OPGs before (T1) and after (T2) the orthodontic treatment were compared and the roots were assigned a respective resorption grade for T2, following the classification mentioned above.

**Figure 3:**
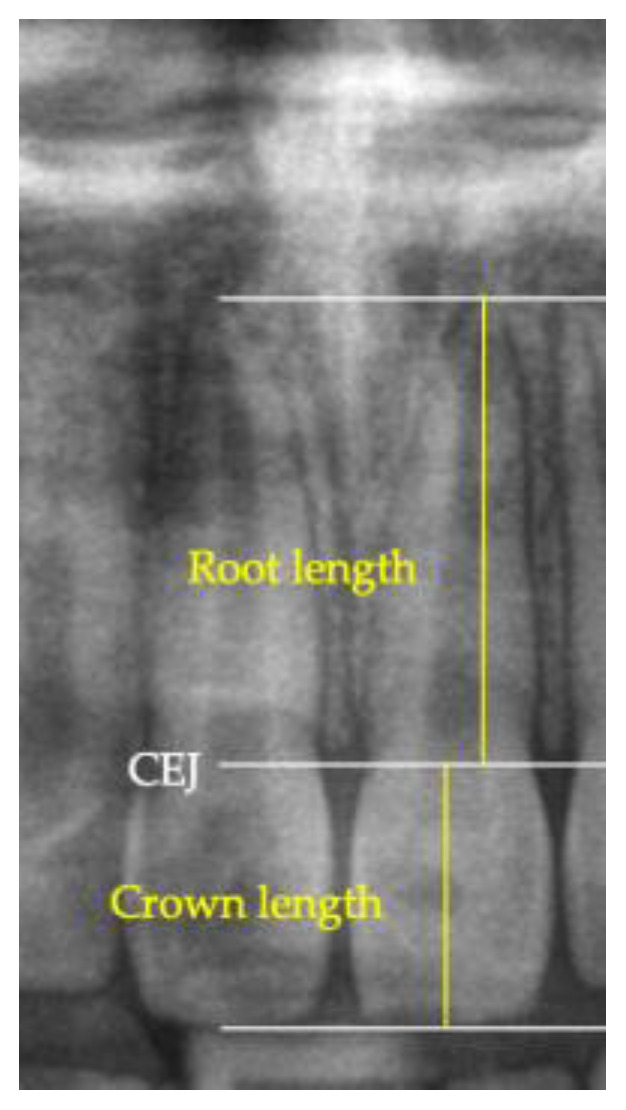
The measurement of crown and root length of tooth 21 on the panoramic radiograph.

The CRR measurement was conducted as follows: Initially, the lengths of both the crowns and roots of each tooth were measured, and subsequently, they were divided to obtain the CRR. This procedure was performed for both T1 and T2, allowing for a comparison of these values. The incisor examination involved defining the crown length from the incisal edge to the horizontal of the mesial and distal cementoenamel junction (CEJ), forming parallel lines. Using a perpendicular, the distance between these parallels was measured, providing the crown length. For the root length, measurements were taken from the horizontal of the mesial and distal CEJ vertically to the root tip. The molar examination involved defining the crown length from the horizontal connection of the mesial and distal cusp tip to the CEJ. The measurement of the root length was analogous to the single-rooted teeth. A straight line was measured that ran perpendicular to the CEJ to the root tip for each root, both mesial and distal (Figure 3).

With these evaluations, the external apical root resorption (EARR) could be calculated using the following formula according to Yi et al. [11]:

EARR (%) = [1 − (CRR after treatment/CRR before treatment)] × 100

The formula can yield positive or negative values, where positive values indicate a presumed lengthening of the root, while negative values indicate a presumed shortening of the root.

The statistical analysis was performed using the BiAS software version 11.10 (BiAS, Epsilon Verlag, Hof, Germany) and R software (R Core Team, 2017).

This study included 57 patients, treated between 2011 and 2018, with a total of 570 teeth and 684 roots analyzed. The data were collected first, and a post hoc power analysis was performed using an effect size of 0.178 and sample sizes of 20 and 37 patients per group, yielding a statistical power of 0.79.

Spearman’s correlation analysis was used to assess the relationship between age, treatment duration, and EARR. Categorical factors such as treatment group, gender, root morphology, and tooth group were evaluated using the Wilcoxon Mann–Whitney U test. The Chi-square test was utilized for nominal scaled variables. Given that each patient contributed 10 analyzed teeth, leading to 10 measurements per patient, the data exhibited interdependencies. To address this, the mixed-model analysis incorporated both fixed and random effects to appropriately account for these dependencies. The statistical significance was set at *p* < 0.05.

## 3. Results

A total of 570 teeth from 57 patients were examined, which ultimately corresponds to 684 roots (Table 1). Overall, the EARR ranged from −42.7% to 36.1% with an average of −5.14% ± 12.27. Although the distribution is approximately symmetrical, the majority is still in the negative range, which represents a root resorption within the treatment period. The positive values could possibly be due to the changes in the axial inclination of the teeth post treatment, which let roots of these teeth appear longer on the panoramic images. Other possible reasons include imaging distortions.

The vast majority (456 roots) had a root-resorption grade of 0 followed by grade 1 with 26.6%. Grade 2 was detected in 6.1% of the roots. Grade 3 and 4 represented a much lower prevalence (0.3% each) compared to grades 0, 1, and 2.

The Spearman correlation was used to evaluate the correlation between age and EARR. With a value of *p* = 0.20, no correlation existed. There was also no correlation between age and the grading according to Levander and Malmgren (*p* = 0.93). With *p* = 0.49, there was no correlation between treatment duration and EARR. The same goes for the grading according to Levander and Malmgren, with *p* = 0.15.

EARR in group 1 ranged from −41.25% to 20.55%, with an arithmetic mean of −2.89% (SD = 11.01). For group 2, the EARR ranged from −42.7% to 36.09%. Here, the average was −4.85% (SD = 11.03). However, there were no significant differences between the two groups regarding the variable EARR (*p* = 0.89). There were also no significant differences between the two groups regarding the grading according to Levander and Malmgren (*p* = 0.56).

The mean EARR for females was −5.84% with a minimum of −42.70% and a maximum of 22.96% (SD = 10.75). The mean EARR for males was −3.92% with a minimum of −40.58% and a maximum of 36.9% (SD = 11.20). The results of the Wilcoxon Mann–Whitney U test revealed that, with a *p*-value of 0.09, there were no statistically significant differences observed between male and female patients in relation to EARR. Regarding the grading according to Levander and Malmgren, there were also no significant differences between the two sexes (*p* = 0.61).

**Figure 4:**
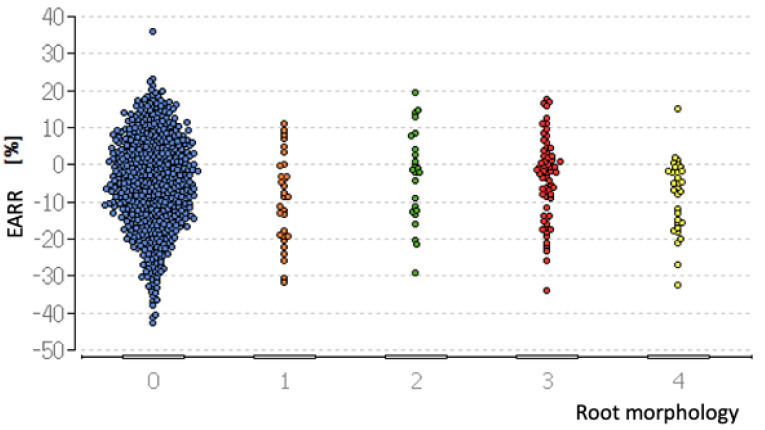
The distribution of external apical root resorption (EARR) stratified based on the root morphology grading system.

The normal root shape according to Levander and Malmgren was the most frequently occurring one with 78.2%, and was followed by the root shape with an apical bend with 9.4%. The remaining root shapes were distributed relatively homogeneously. The pipette-shaped and short root followed, with 4.5% and 4.4%, respectively, and were thus slightly more common than the blunt root shape with 3.5%. The evaluation showed no significant influence of the root morphology regarding EARR (*p* = 0.79). However, there was a significant correlation between the grading according to Levander and Malmgren and the classification of the root morphology (*p* = 0.001) (Figure 4). The short root shape and the pipette-shaped root had a significant influence on the grading according to Levander and Malmgren compared to the normal root shape.

The average resorption of the maxillary anterior teeth was −8.91%. The EARR ranged from −41.3% to 21.3% (SD = 10.71). In contrast, for the mandibular anterior teeth, the mean EARR was −7.21% and the values overall ranged from −42.7% to 36.09% (SD = 12.90). The mandibular molars had an average of −0.56%. The values ranged from −35.62% to 23.8% (SD = 11.05). There was no significant difference between the maxillary anterior teeth and mandibular anterior teeth regarding the EARR (*p* = 0.09). The same applied to the grading according to Levander and Malmgren. The different treatment methods had no significant influence on the individual tooth groups.

## 4. Discussion

After evaluating various approaches for analyzing the available materials, we opted to utilize the CRR calculation method proposed by Yi et al. [11]. This particular formula was chosen because it circumvents the use of direct millimeter measurements and instead centers on the CCR, a measurable quantity. Because OPGs offer only a 2-dimensional image and lack a genuine 3-dimensional representation, it becomes imperative to account for possible variations such as magnifications, contractions, or distortions of the entities being assessed. Consequently, using absolute measurements would not effectively capture the true dimensions depicted in OPGs. Differing from other research that employed the familiar Linge and Linge formula involving millimeter measurements, our study consciously avoided adopting this method [12].

Mirabella et al. [13] conducted research on resorption values below 1.5 mm, while Linge and Linge [12], as well as Sameshima [14], carried out analyses similar to the present study. Likewise, Nigul’s investigation into the effects of orthodontic forces on anterior roots yielded a comparable resorption value of 1.5 mm [15]. However, their studies did not involve the assessment of OPGs; instead, they utilized periapical single-tooth images and measurements were conducted using a ruler. Our choice of imaging modality (OPG instead of periapical radiographs) was based on the study’s focus on accessibility and real-world orthodontic practice. The use of a digital ruler in our study ensured more consistent and accurate measurements compared to manual methods. By standardizing the measuring tool, we minimized operator-induced variability.

Yi et al. determined resorption values to be 5.13% with a standard deviation of 2.81%, which is slightly less than the resorption value observed in the current study. This difference could be due to the fact that Yi et al. limited their examination to the maxillary and mandibular anterior teeth, whereas our sample included the lower first molars as well [11].

Göz and Rakosi highlighted that the majority of orthodontically induced apical resorptions were categorized within grade 1 to 2, a finding that aligns with the observations made in the current study [16].

Most studies do not see any relationship between age and the extend of EARR [17,18,19,20,21,22,23]. In the present study, age can also not be represented as a significant influencing factor. Various authors tend to assert that the crucial factor influencing the degree of root resorption is not necessarily age, but rather the initial state of the periodontium at the commencement of orthodontic treatment [19,20,24]. However, this perspective may be linked to the prevalence of children among orthodontic patients. Similarly, in this study, the average age was 12 years and patients in this age range commonly exhibit healthy periodontal conditions, whereas periodontal issues are more frequently observed after the age of 35–40 years [24,25]. Nonetheless, this particular age cohort constitutes a minority within the field of orthodontics, rendering it challenging to definitively establish a direct link between advanced age, periodontal disease and heightened root resorption. Nevertheless, there appears to be a definitive connection between pre-existing periodontal conditions and a compromised periodontium, which subsequently leads to an escalated occurrence of root resorption due to orthodontic treatment. According to Linge and Linge [26] and supported by Reitan, the magnitude of root resorption tends to decrease when orthodontic treatment is initiated at an earlier stage [26,27]. Their hypothesis is grounded in the idea that younger patients are more adaptable to occlusal and functional modifications compared to older patients.

No significant correlation between the duration of treatment and EARR could be established. The viewpoints in the existing literature diverge on this particular aspect. Zahrowski et al., for instance, identified a higher likelihood of more pronounced EARR with extended and more comprehensive orthodontic treatment durations [28]. Many other authors concurred with this observation [19,29,30,31,32,33,34,35]. Lund, Grondahl, and Hansen et al. could not agree with this assumption [36]. They saw no connection between the duration of treatment and the severity of the orthodontically induced EARR. The consensus in the literature was somewhat weaker here [20,37,38,39,40]. These contrasting perspectives can be linked to the range of treatment methods used. For minor misalignments, shorter treatment durations are often sufficient. However, for more substantial misalignments, shorter treatment periods might involve the application of stronger forces. In cases where these forces exceed the body’s natural tolerance, EARR can occur. Consequently, even in instances of a brief treatment duration, a greater degree of root resorption can manifest.

Linge and Linge published that fixed appliances lead to greater external apical root resorption than removable appliances [26]. Other authors also examined that removable appliance lead to less root resorption than fixed appliances [41,42,43]. Preoteasa et al. compared the root resorptions that resulted from removable and fixed appliances in their study [44]. Root resorptions due to removable appliances could not be found. However, root resorption occurred in 96% of patients with a fixed appliance. In the present study, there were no significant differences between the two treatment groups. The average EARR in both groups was about −5.14%. In our study, flexible CuNiTi archwires were employed during the majority of the treatment period, while a single stainless steel archwire was used only briefly, for about 4–6 weeks toward the end of treatment. The prolonged use of flexible archwires, which exert gentler and more continuous forces, combined with the limited use of stiffer stainless steel wires, may account for the lower levels of EARR observed in our study and the lack of significant differences between the groups.

This study did not find a link between gender and EARR. This observation is further supported by recent literature sources [14,17,38,45,46,47]. In some earlier and less extensive studies, notable differences in root resorption between the two genders were observed. Certain researchers suggested that root resorption was more prevalent and even more severe in females [48,49,50]. This perspective included the work of Kjaer, who introduced the well-known “Kjaer characteristics” [51]. As such, being female is considered one of these “Kjaer traits,” and it might offer some predictive value for the extent of EARR.

A correlation between EARR and abnormal root shape such as pipette anatomy was demonstrated in this study. This finding has also been supported in the literature [14,52].

However, the relationship between the grading based on Levander and Malmgren’s criteria and root shape was indeed evident. The short and pipette-shaped root forms exhibited a noteworthy impact on the grading, especially when compared to the normal root shape. This observation finds confirmation in Kjaer’s work [51]. In 1995, Kjaer expanded the Kjaer characteristics to encompass pipette-shaped roots, which are indicative of morphological factors influencing root resorption [51]. According to Kjaer, teeth with pipette-shaped roots are more susceptible to root resorption than those with a standard root shape. Similarly, Levander et al. discovered that roots featuring apical bends, bluntness, or a pipette-shaped form are more prone to root resorption [9,35,53]. On the contrary, Van Parys et al. found no association between pipette-shaped roots in the maxillary incisors and a higher risk of orthodontic EARR [53].

The limitations of 2D imaging, such as orthopantomograms (OPGs), are well documented, as they cannot detect apical root bends or accurately represent tooth inclinations. Roots may appear shorter or longer due to inclination changes rather than actual resorption. Additionally, resorptive changes on non-apical surfaces, such as lateral or lingual aspects, cannot be visualized using OPGs. Despite these drawbacks, 2D imaging remains the standard of care in orthodontics due to its accessibility and lower radiation exposure. While cone-beam computed tomography (CBCT) offers superior accuracy for assessing EARR, its higher radiation dose and lack of coverage by public insurance systems in countries like Germany limit its routine use [54]. Beyond CBCT, finite-element analysis studies may provide innovative, non-radiographic approaches for a more comprehensive understanding of root resorption mechanics. Alternatively, combining OPG with lateral cephalometric X-rays could offer additional insights into root-resorption measurement by providing a view in a second dimension.

## 5. Conclusions

The study’s findings suggest that the two-phase orthodontic treatment does not increase the risk of EARR compared to one-phase therapy significantly. Some degree of root resorption occurred as a result of orthodontic treatment in both groups. Notably, abnormal root forms were identified as influential factors that could help predict the likelihood of root resorption following orthodontic treatment.

## Figures and Tables

**Table 1:** Descriptive statistics of crown–root ratio (CRR) at T1 (pre treatment) and T2 (post treatment) and external apical root resorption (EARR) stratified by tooth number and group (1: patients undergoing one-phase of therapy with fixed orthodontic appliances; 2: patients undergoing two-phase treatment, including removable functional appliances followed by fixed orthodontic treatment).

Group	Tooth	CRR (T1)		CRR (T2)		EARR	
		Mean ± SD	Median (IQR)	Mean ± SD	Median (IQR)	Mean ± SD	Median (IQR)
1	11	63.84 ± 9.64	62.3 (11.27)	71.64 ± 8.62	70.5 (11.7)	−12.89 ± 7.72	−12.82 (13.81)
	12	59.17 ± 6.14	59.85 (9.05)	65.16 ± 7.84	63.25 (9.0)	−9.78 ± 12.03	−7.6 (11.53)
	21	63.55 ± 8.52	62.45 (8.25)	70.53 ± 8.09	69.25 (8.83)	−11.44 ± 7.21	−11.72 (7.96)
	22	43.88 ± 5.79	44.5 (8.38)	42.52 ± 4.0	42.05 (6.32)	−9.14 ± 12.43	−10.47 (18.4)
	31	48.69 ± 5.71	50.2 (6.77)	49.67 ± 6.43	48.9 (7.5)	−2.65 ± 13.1	−0.04 (16.86)
	32	44.74 ± 6.65	43.0 (9.33)	46.8 ± 5.38	44.9 (8.65)	−6.13 ± 15.55	−4.99 (18.21)
	41	47.74 ± 6.16	46.5 (9.12)	51.92 ± 7.92	50.8 (8.23)	−8.91 ± 10.24	−10.84 (13.7)
	42	45.44 ± 5.75	46.5 (6.9)	47.44 ± 5.16	45.65 (6.2)	−5.19 ± 10.64	−2.56 (17.06)
	36 d	45.94 ± 6.22	46.2 (6.62)	44.87 ± 4.42	43.85 (6.53)	−3.53 ± 10.07	0.23 (14.61)
	36 m	45.94 ± 6.22	46.2 (6.62)	44.87 ± 4.42	43.85 (6.53)	−1.31 ± 8.21	0.45 (13.36)
	46 d	45.94 ± 6.22	46.2 (6.62)	44.87 ± 4.42	43.85 (6.53)	1.32 ± 11.08	−0.12 (17.04)
	46 m	43.88 ± 5.79	44.5 (8.38)	42.52 ± 4.0	42.05 (6.32)	2.01 ± 11.99	4.59 (15.72)
2	11	64.69 ± 6.8	64.5 (8.8)	69.4 ± 7.68	68.5 (9.6)	−7.53 ± 8.17	−6.51 (10.86)
	12	59.48 ± 5.85	59.8 (5.9)	64.56 ± 9.78	61.8 (8.7)	−8.42 ± 9.92	−7.62 (12.4)
	21	65.08 ± 6.02	64.6 (7.0)	68.43 ± 7.89	67.7 (10.2)	−5.42 ± 10.52	−4.19 (12.49)
	22	45.26 ± 3.94	45.4 (5.6)	44.55 ± 4.61	44.4 (6.9)	−9.81 ± 14.34	−1.93 (21.77)
	31	47.88 ± 4.81	48.4 (5.5)	50.84 ± 5.25	51.2 (5.8)	−6.71 ± 10.95	−6.09 (15.47)
	32	45.82 ± 4.73	46.2 (5.5)	47.35 ± 5.13	47.2 (7.1)	−4.22 ± 14.06	−4.51 (9.21)
	41	48.93 ± 6.14	49.1 (7.1)	53.19 ± 6.85	53.3 (9.1)	−9.53 ± 13.95	−6.82 (17.85)
	42	45.28 ± 4.15	45.5 (5.6)	50.3 ± 5.23	49.1 (9.1)	−11.62 ± 12.34	−10.55 (16.89)
	36 d	47.65 ± 4.41	47.3 (4.7)	47.89 ± 5.51	47.8 (8.4)	−2.81 ± 11.65	−2.43 (15.01)
	36 m	47.65 ± 4.41	47.3 (4.7)	47.89 ± 5.51	47.8 (8.4)	−0.18 ± 12.49	1.74 (13.69)
	46 d	47.65 ± 4.41	47.3 (4.7)	47.89 ± 5.51	47.8 (8.4)	−0.81 ± 10.67	0.21 (16.0)
	46 m	45.26 ± 3.94	45.4 (5.6)	44.55 ± 4.61	44.4 (6.9)	1.19 ± 10.76	3.49 (18.24)

## Data Availability

The data can be provided upon request to the corresponding author.
